# Identification and Characterisation of an Iron-Responsive Candidate Probiotic

**DOI:** 10.1371/journal.pone.0026507

**Published:** 2011-10-19

**Authors:** Jennifer R. Bailey, Christopher S. J. Probert, Tristan A. Cogan

**Affiliations:** 1 Mucosal Microbiology, School of Veterinary Sciences, University of Bristol, Bristol, United Kingdom; 2 School of Clinical Science, University of Bristol, Bristol, United Kingdom; University of California Merced, United States of America

## Abstract

**Background:**

Iron is an essential cofactor in almost all biological systems. The lactic acid bacteria (LAB), frequently employed as probiotics, are unusual in having little or no requirement for iron. Iron in the human body is sequestered by transferrins and lactoferrin, limiting bacterial growth. An increase in the availability of iron in the intestine by bleeding, surgery, or under stress leads to an increase in the growth and virulence of many pathogens. Under these high iron conditions, LAB are rapidly out-competed; for the levels of probiotic bacteria to be maintained under high iron conditions they must be able to respond by increasing growth rate to compete with the normal flora. Despite this, iron-responsive genera are poorly characterised as probiotics.

**Methodology/Principal Findings:**

Here, we show that a panel of probiotics are not able to respond to increased iron availability, and identify an isolate of *Streptococcus thermophilus* that can increase growth rate in response to increased iron availability. The isolate of *S. thermophilus* selected was able to reduce epithelial cell death as well as NF-κB signalling and IL-8 production triggered by pathogens. It was capable of crossing an epithelial cell barrier in conjunction with *E. coli* and downregulating Th1 and Th17 responses in primary human intestinal leukocytes.

**Conclusions/Significance:**

We propose that an inability to compete with potential pathogens under conditions of high iron availability such as stress and trauma may contribute to the lack of efficacy of many LAB-based probiotics in treating disease. Therefore, we offer an alternative paradigm which considers that probiotics should be able to be competitive during periods of intestinal bleeding, trauma or stress.

## Introduction

The benefit of consuming specific strains of bacteria was first proposed by Elie Metchnikoff. He suggested that since lactic acid bacteria can prevent putrefaction of stored food, they may also benefit the gastrointestinal tract; Bulgarian bacillus (later identified as *Lactobacillus delbruickii* subspecies *bulgaricus*) isolated from a fermented milk product was of particular interest. Metchnikoff proposed it was the optimal strain to consume because of its ability to produce large amounts of lactic acid with little succinic or acetic acid; its ability to coagulate milk rapidly; and the lack of alcohol and acetone produced [1]. Interest in probiotics waned with the advent of antibiotics. However, with the emergence of antibiotic-resistant bacteria, there is renewed interest in probiotic bacteria, now defined as “live microorganisms which when administered in adequate amounts confer a health benefit on the host” [2]. Following on from Metchnikoff's work, candidate probiotics, in particular *Lactobacillus* spp., have been trialled in the treatment of a number of diseases.

Some *Lactobacillus* spp. have been shown to reduce symptoms in allergic rhinitis [3] and atopic eczema [4], [5]. Specifically, *L. casei* Shirota may reduce the severity of allergic rhinitis sufferers by reducing antigen-induced IL-5, IL-6 and IFN-γ, as well as specific IgE [3]. Meanwhile, *L. rhamnosus* (strain GG ATCC 53103), *L. reuteri* (ATCC 55730) and *L. paracasei* (strain F19) have been shown to decrease the incidence of eczema in cohorts of children [6], [7], [8], [9], [10].

*Lactobacillus* spp. have also been successful in the treatment of acute infectious diarrhoea in children [11] and prevention of traveller's diarrhoea [12] and antibiotic-associated diarrhoea (AAD) [13], but not Crohn's disease [14]. In contrast, two probiotic preparations which are not based upon *Lactobacillus* spp., VSL#3 and *Escherichia coli* Nissle 1917, have shown promise in the treatment of inflammatory bowel disease (IBD) [15], [16], [17], [18]. Crohn's disease is considered to be a response to an environmental trigger in a genetically susceptible host. The environmental trigger is thought to be bacteria and current research is now focuses on adherent-invasive *E. coli* (AIEC) [19]. If Crohn's disease is triggered by bacteria then it is an attractive candidate for treatment with a probiotic which could either outcompete the bacteria or divert the immune response in order to prevent the uncontrolled inflammation which is a characteristic feature of IBD. However, in order for any organism to carry out either of these functions it must be able to survive and compete within this challenging environment.

The lactic acid bacteria (LAB) are unusual organisms in that they do not appear to have a requirement for iron [20], [21], [22] whilst maintaining a high demand for manganese [23]. In the human body, iron is sequestered by the transferrins and lactoferrin [24]. Iron sequestration is considered the primary factor limiting bacterial growth rate in the body. An increase in the availability of iron in the intestine by dietary supplementation, intestinal bleeding, surgery, trauma or under stress, will lead to an increase in the abundance of many bacterial species. This is mediated by a greater availability of free iron, or by the presence of noradrenaline, which unloads iron from chelators and can supply it to some species of bacteria [25], [26]. Under these high-iron conditions, LAB are rapidly outcompeted as other species increase their growth rate in response to iron availability and predominate. Thus, for the levels of probiotic bacteria to be maintained under high iron conditions they must be able to respond to this element by increasing growth rate in order to compete with the normal flora. We propose that an inability to compete with potential pathogens under conditions of stress and trauma may contribute to the lack of efficacy of many LAB-based preparations in treating disease.

Here, we show that the majority of LAB do not respond to noradrenaline-mediated iron availability by increasing growth rate. We have identified species of bacteria that can increase growth rate under these conditions, and using a rational selection process we have found an isolate of *S. thermophilus* with probiotic potential, based on functionality in *in vitro* models. We propose an alternative paradigm to the traditional LAB spp. probiotics, which considers that probiotics should be able to be active and functional during periods of intestinal bleeding, trauma or stress.

## Results

### Growth of bacteria with noradrenaline

Noradrenaline can remove iron from chelators and supply it to bacteria. A number of LAB were cultured with and without noradrenaline to determine whether they were capable of responding to it, or the iron provided by it (Table 1). While the addition of noradrenaline had no effect on most LAB studied, two strains significantly increased their growth in response to it: *L. acidophilus* ASF360 increased its growth more than 7-fold at 48 hours and *S. thermophilus* NCIMB 41856 increased growth at all time points studied, with a maximum of an almost 5-fold increase at 48 hours (Table 1). These two strains were chosen for further characterisation of their probiotic potential, alongside *E. coli* Nissle 1917 which has been used for the treatment of IBD [15].

**Table 1:** Response of lactic acid bacteria to noradrenaline.

	24 Hours	48 Hours	72 Hours
***L. bulgaricus*** **JB005**	1.227±0.215	1.328±0.279	1.266±0.125
***L. casei*** **JB006**	0.848±0.144	1.191±0.180	1.440±0.017
***L. casei*** **JB008**	1.140±0.074	1.035±0.125	1.357±0.446
***L. acidophilus*** **ASF360**	1.285±0.179	7.725±2.519 *	2.375±0.937
***L. salivarius*** **ASF361**	1.021±0.128	0.865±0.175	1.187±0.173
***L. plantarum*** **JB012**	1.014±0.170	0.943±0.115	1.112±0.250
***L. helveticus*** **JB011**	0.999±0.114	1.003±0.097	1.004±0.214
***B. animalis*** **JB007**	0.998±0.313	1.246±0.398	1.037±0.326
***B. bifidum*** **JB009**	1.175±0.134	1.209±0.172	1.190±0.169
***S. thermophilus*** **JB004**	0.986±0.296	1.374±0.109	1.576±0.439
***S. thermophilus*** **NCIMB 41856**	1.700±0.242 *	4.798±0.868 *	3.869±1.954 *

* Indicates p≤0.05 when compared to the mean value for each time point.

### Proliferation and death of epithelial cells

**Figure 1:**
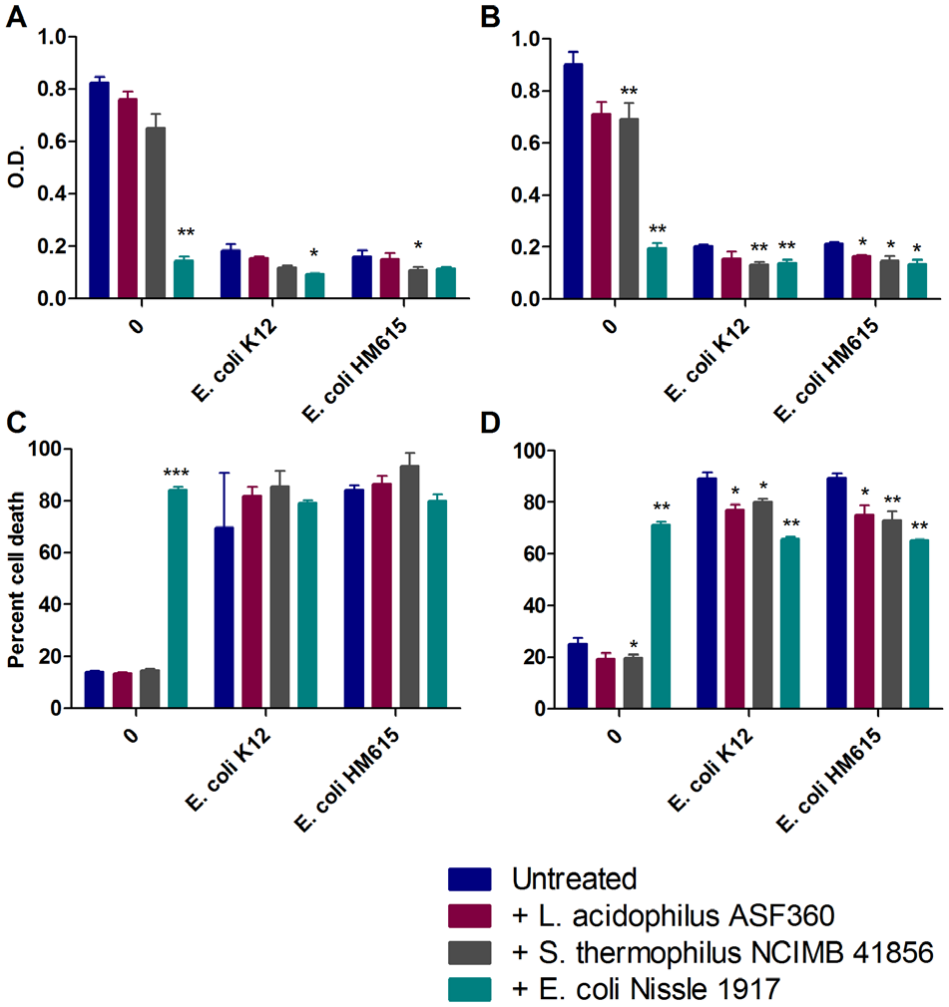
Probiotic effect on proliferation and death of epithelial cells in response to pathogenic *E. coli*. A) Proliferation of T84 cells; B) proliferation of Caco-2 cells; C) death of T84 cells; D) death of Caco-2 cells. Results are shown from 3 replicate experiments and are expressed as mean + S.E.M. * p≤0.05, ** p≤0.01 and *** p≤0.001.

Increased turnover of epithelial cells is a common response to infection therefore T84 and Caco-2 adenocarcinoma cells were incubated with the potential probiotics *L. acidophilus* ASF360, *S. thermophilus* NCIMB 41856 and *E. coli* Nissle 1917 to determine their effect on the proliferative or apoptotic cellular response to pathogenic *E. coli* strains, K12 and the Crohn's disease-associated AIEC strain HM615 [27]. All *E. coli* strains, including *E. coli* Nissle 1917, reduced the proliferation of T84 epithelial cells: *E. coli* K12 reduced proliferation by 78% compared to untreated cells (p = 0.002); AIEC HM615 reduced proliferation by 80% (p = 0.0001); and *E. coli* Nissle 1917 by 82% (p = 0.001) (Figure 1A). A similar reduction in proliferation of Caco-2 cells was seen following *E. coli* treatment: *E. coli* K12 induced a reduction of 77% compared to untreated cells (p = 0.003); AIEC HM615 induced a reduction of 76% (p = 0.003); and *E. coli* Nissle 1917 of 78% (p = 0.004) (Figure 1C). Simultaneously, cell death was increased in both T84 and Caco-2 cells; *E. coli* K12 induced a 254% increase in cell death in Caco-2 cells (p = 0.0005); AIEC HM615 induced a 498% increase in death in T84 cells (p = 0.0003) and a 254% increase in Caco-2 cell death (p = 0.001); *E. coli* Nissle 1917 induced a 498% increase in T84 cell death (p<0.0001) and a 218% increase in Caco-2 cell death (p = 0.001) (Figure 1D). *S. thermophilus* NCIMB 41856 reduced the proliferation of Caco-2 cells treated with *E. coli* K12 or AIEC HM615 by 35% (p = 0.003) and 31% (p = 0.05), respectively; while *E. coli* Nissle 1917 reduced proliferation induced by *E. coli* K12 by 32% (p = 0.007) and 37% in response to AIEC HM615 treatment (p = 0.03) (Figure 1B). In addition, *L. acidophilus* ASF360 further reduced proliferation of Caco-2 cells treated with AIEC HM615 by 22% (p = 0.02) (Figure 1B). *S. thermophilus* NCIMB 41856 reduced proliferation of T84 cells treated with AIEC HM615 and *E. coli* Nissle 1917 by 33% (p = 0.03) and 48% (p = 0.03), respectively (Figure 1A). Importantly, all three probiotic strains reduced death of Caco-2 cells following challenge with both *E. coli* K12 and HM615: *L. acidophilus* ASF360 reduced death of epithelial cells by 14% (p = 0.04) following *E. coli* K12 treatment and 16% following infection with AIEC HM615 (p = 0.03); *S. thermophilus* NCIMB 41856 reduced death of epithelial cells following *E. coli* K12 and AIEC HM615 treatment by 10% (p = 0.01) and 18% (p = 0.007), respectively; *E. coli* Nissle 1917 reduced death of epithelial cells following *E. coli* K12 and treatment by 26% (p = 0.006) and 27% (p = 0.003), respectively (Figure 1D). In addition, *S. thermophilus* NCIMB 41856 was able to reduce proliferation and cell death of untreated Caco-2 cells by 24% (p = 0.008) and 22% (p = 0.02) respectively (Figure 1B and D).

### Induction of NF-κB and IL-8

**Figure 2:**
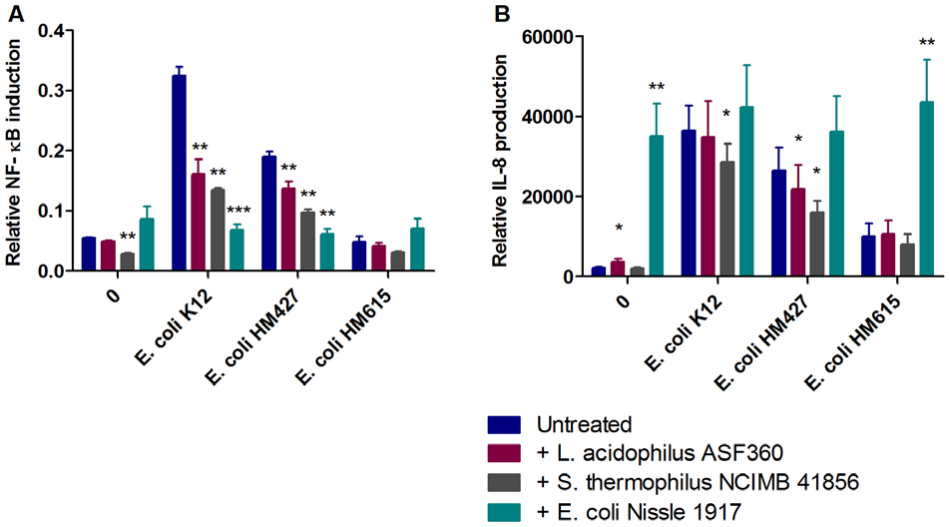
Effect of probiotics on NF-κB (A) and IL-8 (B) response to pathogen. Results are shown from 6 replicate experiments and are expressed as mean + S.E.M. * p≤0.05, ** p≤0.01 and *** p≤0.001.

Monolayers of Caco-2 and T84 cells were treated with the three potential probiotic bacterial strains in combination with pathogenic *E. coli* strains in order to determine the effect of probiotic on either of these pro-inflammatory signalling events. NF-κB signalling in Caco-2 cells was upregulated by 491% (p = 0.002) compared to controls following infection with *E. coli* K12 and by 247% (p = 0.002) following AIEC HM427 treatment. However, this induction was reduced by the addition of all three probiotic strains: *L. acidophilus* ASF360 reduced the NF-κB response to *E. coli* K12 by 50% (p = 0.006) and AIEC HM427 by 28% (p = 0.007); *S. thermophilus* NCIMB 41856 reduced the NF-κB response to *E. coli* K12 by 58% (p = 0.002) and AIEC HM427 by 49% (p = 0.005); and *E. coli* Nissle 1917 reduced the NF-κB response to *E. coli* K12 by 79% (p = 0.0005) and AIEC HM427 by 68% (p = 0.004) (Figure 2A). *S. thermophilus* NCIMB 41856 also reduced NF-κB signalling by 48% in untreated cells (p = 0.003) (Figure 2A). Following NF-κB signalling, IL-8 production was increased by 1566% in response to *E. coli* K12 (p = 0.001), 1104% in response to AIEC HM427 (p = 0.004) and 363% in response to HM615 (p = 0.02), as well as a 1498% increase following addition of *E. coli* Nissle 1917 (p = 0.004). The IL-8 response to *E. coli* K12 was reduced by 22% following addition of *S. thermophilus* NCIMB 41856 (p = 0.02) and the response to AIEC HM427 was reduced by both *L. acidophilus* ASF360 and *S. thermophilus* NCIMB 41856 by 17% (p = 0.02) and 39% (p = 0.02) respectively (Figure 2B).

### Maintenance of epithelial barrier integrity

**Figure 3:**
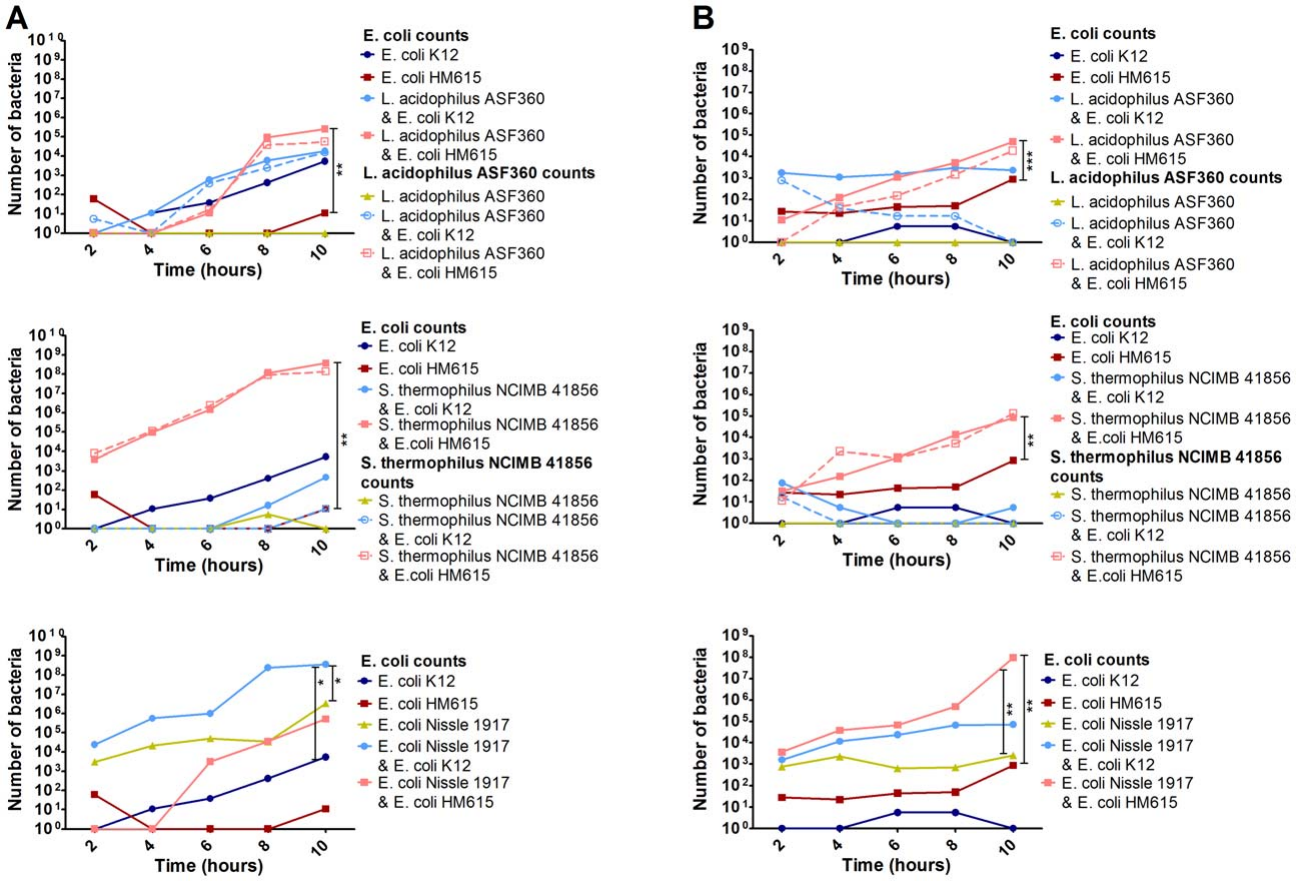
Translocation of bacteria through a T84 (A) or Caco-2 (B) epithelial cell monolayer. Results are shown from 3 replicates and are expressed as means. * p≤0.05, ** p≤0.01 and *** p≤0.001.

To determine the effect of our probiotic strains on epithelial barrier integrity, Caco-2 and T84 cells were grown in a Transwell system and challenged with *E. coli* K12 or AIEC HM615 in combination with each of the potential probiotics; TEER and bacterial translocation were measured. Both Caco-2 and T84 cells formed stable monolayers after 8–10 days. Neither *L. acidophilus* ASF360 nor *S. thermophilus* NCIMB 41856 had any effect on TEER alone. However, *S. thermophilus* NCIMB 41856 blocked the passage of *E. coli* K12 through the monolayer, a phenomenon not seen with *L. acidophilus* ASF360 which enhanced the passage of *E. coli* K12 across the barrier (Figure 3). In addition, *S. thermophilus* NCIMB 41856 reduced the response to *E. coli* K12, by increasing TEER, in a way that *L. acidophilus* ASF360 did not; when *S. thermophilus* NCIMB 41856 and *E. coli* K12 were added to the monolayer simultaneously, an increase in TEER was seen (peaking at 33% in Caco-2 cells at 6 hours and 30% in T84 cells at 10 hours compared to K12 stimulated cells), whereas *L. acidophilus* ASF360 and *E. coli* K12 together caused a decrease in TEER (peaking at 56% in Caco-2 cells at 8 hours and 11% in T84 cells at 6 hours compared to K12 stimulated cells) (Figure S1). While AIEC HM615 alone slowly migrated through the monolayer, both *L. acidophilus* ASF360 and *S. thermophilus* NCIMB 41856 appeared to interact with this strain and facilitate its migration across the epithelial monolayer. In this situation, translocation of *L. acidophilus* ASF360 and *S. thermophilus* NCIMB 41856 was increased until these probiotic strains were present in equal numbers to the pathogenic strain (Figure 3). The potentially probiotic *E. coli* strain Nissle 1917, similarly to *L. acidophilus* ASF360 and *S. thermophilus* NCIMB 41856, had no effect on TEER (Figure S1) but it was able to translocate quickly without the need to interact with pathogenic *E. coli* strains (Figure 3).

### Maintenance of tight cell junctions

**Figure 4:**
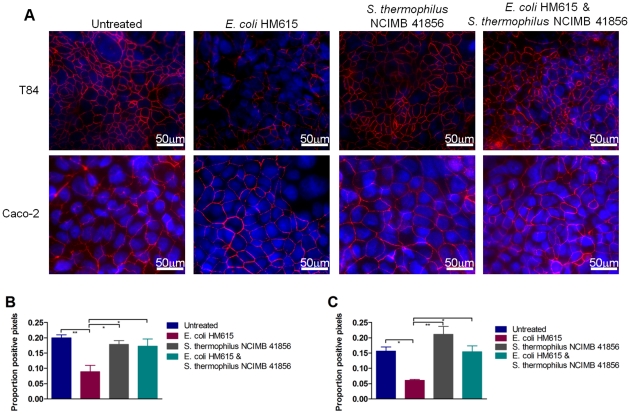
Maintenance of tight cell junctions by probiotics. Expression of occudin (red) in epithelial cell monolayers (A). Proportion of pixels positive for occludin staining relative to entire field of view in T84 (B) and Caco-2 cells (C). Results are expressed as mean+S.E.M. * p≤0.05 and ** p≤0.01.

Caco-2 and T84 cells were grown in a Transwell system and infected with AIEC HM615; *S. thermophilus* NCIMB 41856 was added to determine its effect on the tight cell junction protein occludin. AIEC HM615 caused the breakdown of tight cell junctions in Caco-2 and T84 monolayers, illustrated by decreased occludin (61% and 56% respectively) (Figure 4). AIEC HM615 also caused a 24% decrease in nuclear staining of T84 cells (data not shown), indicating that it was inducing cell death. The addition of *S. thermophilus* NCIMB 41856 to the monolayers in conjunction with AIEC HM615 prevented AIEC-induced tight cell junction breakdown and cell death; levels of nuclear and occludin staining were unchanged from control monolayers (Figure 4).

### Skewing of effector T cell responses

**Figure 5:**
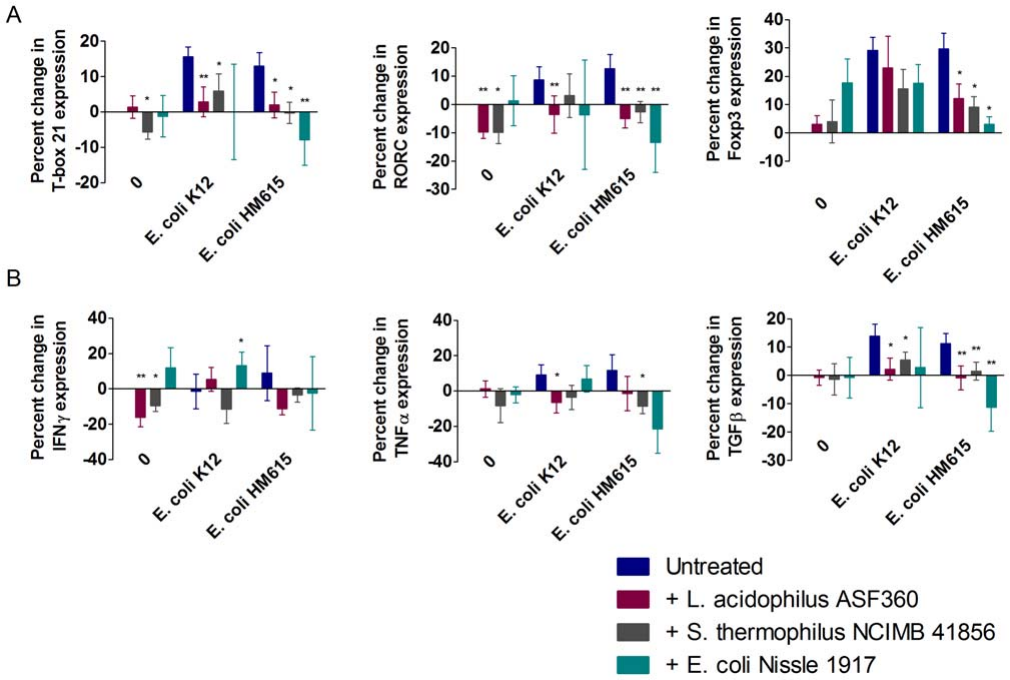
Effect of probiotics on the adaptive immune response. Percent change in expression of T-box21 (Th1), RORC (Th17) and Foxp3 (Treg) transcription factor mRNA in response to *E. coli* and the effect of probiotics on their expression (A). Percent change in cytokine mRNA levels induced by T cells in response to *E. coli* with and without probiotic (B). Results are expressed as mean±S.E.M. * p≤0.05 and ** p≤0.01.

Leukocytes were isolated from the intestinal lamina propria and challenged with bacterial antigens from *E. coli* K12 and AIEC HM615 to determine the effect of competing probiotic antigens from *L. acidophilus* ASF360, *S. thermophilus* NCIMB 41856 and *E. coli* Nissle 1917. Both *E. coli* K12 and AIEC HM615 induced a Th1 response, indicated by upregulation of mRNA encoding the Th1-specific transcription factor T-box21 in the population of cultured leukocytes (16% (p = 0.003) and 13% (p = 0.007) respectively); this was significantly reduced by the addition of *L. acidophilus* ASF360 or *S. thermophilus* NCIMB 41856. *L. acidophilus* ASF360 reduced the response to *E. coli* K12 and AIEC HM615 by 13% (p = 0.003) and 11% (p = 0.02), respectively. *S. thermophilus* NCIMB 41856 reduced the Th1 response to *E. coli* K12 and AIEC HM615 by 10% (p = 0.03) and 13% (p = 0.04), respectively. *E. coli* Nissle 1917 also downregulated the Th1 response to AIEC HM615 by 21% (p = 0.009). Furthermore, *S. thermophilus* NCIMB 41856 reduced the baseline level of transcription of T-box21 in untreated cells by 6% (p = 0.03). Neither *E. coli* strain induced a significant Th2 response but the Th17-specific transcription factor, RORC, was also upregulated following treatment with *E. coli* K12 or AIEC HM615 antigens (9% and 13% (p = 0.02), respectively). The Th17 response to *E. coli* K12 was reduced by 12% following the addition of *L. acidophilus* ASF360 antigens (p = 0.009) and the response to AIEC HM615 was reduced by the addition of any of the three potential probiotic strains: *L. acidophilus* ASF360 reduced the response by 18% (p = 0.0002), *S. thermophilus* NCIMB 41856 by 15% (p = 0.003) and *E. coli* Nissle 1917 by 26% (p = 0.003). In addition, both *L. acidophilus* ASF360 and *S. thermophilus* NCIMB 41856 were capable of reducing the baseline level of RORC transcription in untreated cells by 10% (p = 0.003 and p = 0.04, respectively). AIEC HM615 also induced a strong Treg response, shown by the upregulation of Foxp3 by 30% (p = 0.0009) in cultured leukocytes. However, this was reduced by the addition of any of the three potential probiotic strains: *L. acidophilus* ASF360 caused an 18% reduction in Foxp3 expression (p = 0.02), *S. thermophilus* NCIMB 41856 a 21% reduction (p = 0.03) and *E. coli* Nissle 1917 a 27% reduction (p = 0.02) (Figure 5A).

### T cell cytokine production

In accordance with the transcription factor data previously described, both *E. coli* K12 and AIEC HM615 induced upregulation of TNFα mRNA in cultured leukocyte populations (9% and 12% respectively), indicating a Th1 response. Despite this, neither *E. coli* K12 or AIEC HM615 induced a significant IFNγ response, although IFNγ mRNA production was increased by 15% when *E. coli* K12 and Nissle 1917 were used in combination (p = 0.04) and decreased by 16% following treatment with *L. acidophilus* ASF360 (p = 0.01) or by 10% following treatment with *S. thermophilus* NCIMB 41856 (p = 0.02) alone. The TNFα response to *E. coli* K12 was reduced by 16% by the addition of *L. acidophilus* ASF360 (p = 0.03) whereas *S. thermophilus* NCIMB 41856 reduced the level of TNFα mRNA produced in response to AIEC HM615 by 20% (p = 0.03) (Figure 5B). As expected, there was no significant change in the production of the Th2-related cytokine IL-4. Although not significant, both *E. coli* strains appeared to increase the expression of IL-4δ2 mRNA, the naturally occurring antagonist of IL-4, further indicating skewing towards a Th1 response. However, evidence for a Th17 response was seen in the increased expression of IL-17 mRNA by 45% (p = 0.04) following treatment with antigens derived from AIEC HM615; this appeared to be reduced by the addition of any of the three probiotic strains, although this was not statistically significant. No significant changes were seen in the expression of IL-10 mRNA, although *E. coli* Nissle 1917 did reduce IL-10 expression by 29% when cultured in combination with AIEC HM615; however HM615 did not induce the expression of IL-10 (Figure S2). The transcription of TGFβ was significantly increased by lamina propria leukocytes by 14% in response to *E. coli* K12 (p = 0.01) and by 11% in response to AIEC HM615 (p = 0.009) treatment. *L. acidophilus* ASF360 and *S. thermophilus* NCIMB 41856 were able to reduce the induction of TGFβ mRNA by *E. coli* K12 by 12% (p = 0.01) and 9% (p = 0.006) to levels comparable to that of control cells. Similarly, *L. acidophilus* ASF360 and *S. thermophilus* NCIMB 41856 also reduced the TGFβ response to AIEC HM615 by 12% (p = 0.04) and 10% (p = 0.009) respectively. *E. coli* Nissle 1917 was also able to reduce the expression of TGFβ mRNA induced by AIEC HM615 by 23% (p = 0.01) (Figure 5B).

## Discussion

The mechanisms through which probiotics have been hypothesised to act are numerous and include an influence on intestinal transit time, competition with pathogens and immunomodulation. It is not clear what effect on the immune system is most desirable. A pro-inflammatory response may be required in order to more effectively clear infection [28], [29], however prolonged NF-κB activation, and subsequent production of IL-8, RANTES and CXCL10, has been implicated in animal models of IBD [30]. In contrast some strains of bacteria have been shown to act specifically on components of the adaptive immune response in order to reduce inflammation and promote regulation. *Faecalibacterium prausnitzii* A2–165 (DSM 17677) is capable of reducing the expression of the pro-inflammatory cytokines IL-12 and IFNγ by PBMCs [31]. Upregulation of regulatory IELs in a mouse model of colitis was induced by two mixtures of probiotics: *L. acidophilus* Bar 13 and *B. longum* Bar 33; *L. plantarum* Bar 10, *S. thermophilus* Bar 20 and *B. animalis* subsp. *lactis* Bar 30 [32]. Furthermore, a combination of *L. acidophilus*, *L. casei*, *L. reuteri*, *B. bifidum* and *S. thermophilus* downregulated Th1, Th2 and Th17 cytokine responses, induced generation of CD4^+^ Foxp3^+^ Tregs and promoted regulatory dendritic cells expressing high levels of the regulatory cytokines IL-10 and TGFβ [33].

LAB have uniquely very low requirements for iron [20], [21], [22]. The increased bioavailability of iron during intestinal bleeding can increase the growth rate and virulence of many gastrointestinal pathogens [34]; under these conditions, LAB can be outcompeted. The results of this work indicate that most LAB cannot respond to increased iron bioavailability (Table 1), with the exception of *Lactobacillus acidophilus* ASF360 and *Strep. thermophilus* NCIMB 41856. One of the principal mechanisms for the action of probiotics is thought to be competitive exclusion of pathogens. These results support the theory that LAB are inefficient during active inflammatory disease because of their inability to compete with pathogens in the presence of iron, due to bleeding or supplementation.

For a probiotic to be effective in treating IBD, we consider that it must be able to effectively compete with pathogens under conditions encountered in the non-healthy intestine. It should also be able to control immune responses to pathogens and restore normality. These are the primary properties that we investigated in this work. Using a rational selection process we have determined that the iron-responsive strain of *S. thermophilus* NCIMB 41856 shows probiotic potential. This *S. thermophilus* NCIMB 41856 performed at least as well, and in many cases, better than, the more widely researched strains of *L. acidophilus* and *E. coli* Nissle 1917. *L. acidophilus* ASF360 showed a growth increase in response to iron, but only after 48 h, with the increase not being sustained after 72 h. In order for an organism to be able to establish itself and compete against pathogens in an iron-rich environment, stimulation of growth should ideally occur within typical intestinal transit time and then be sustained. This organism did not meet this test. In contrast, *E. coli* is known to be able to respond to iron in this manner [34], as is *S. thermophilus* NCIMB 41856 (Table 1); however, whereas *S. thermophilus* NCIMB 41856 promoted an anti-inflammatory response, *E. coli* Nissle 1917 provoked a pro-inflammatory response, consistent with the findings of other groups [35]. It has been hypothesised that activation of epithelial cells, as demonstrated here by the ability of *E. coli* Nissle 1917 to induce an NF-κB and IL-8 response, leads to an increase in innate immune defences, thereby improving barrier function [36]. However, concerns have been raised about the safety of *E. coli* Nissle 1917 as a probiotic, particularly in immunocompromised patients [37].

The results presented here show that our strain of *S. thermophilus* NCIMB 41856 appears to be a promising candidate as a probiotic, at least in our *in vitro* experiments. *S. thermophilus* has largely been overlooked as a probiotic strain of bacteria in favour of *Lactobacillus* or *Bifidobacterium* spp and little work has been done to determine its mode of action. Despite this, it is a constituent of the VSL#3 probiotic preparation, one of the few probiotic therapies to have a significant effect on the treatment of IBD [18]. *S. thermophilus* is also present in the majority of fermented milk products, some of which have been successfully used as therapeutic treatments [38]; however the levels of bacteria are too low to have a probiotic effect [39]. We have found that the beneficial effect exerted by *S. thermophilus* NCIMB 41856 is dose-dependent, with the optimum dose being 1000 times more concentrated than that found in the majority of fermented milk products previously trialled. Few studies have looked into the probiotic effect of *S. thermophilus* alone, however, it was reported that milk fermented with this strain of bacteria was equivalent to proton-pump inhibitors in reducing gastritis induced by non-steroidal anti-inflammatory drugs [40].

We propose that the predominant effect of *S. thermophilus* NCIMB 41856 would be in limiting the pro-inflammatory response initiated by the innate immune system. Furthermore, it acts on the adaptive immune system by modulating the T cell response, promoting regulation and reducing inflammation. The pro-inflammatory response to pathogenic *E. coli* was essentially cancelled out by the addition of *S. thermophilus* NCIMB 41856, returning levels of the varying T cell subsets to those seen under normal conditions in our *ex vivo* system. This was further emphasised by the ability of *S. thermophilus* NCIMB 41856 to reduce the transcription of mRNA encoding TNFα in response to AIEC. As previously mentioned, AIEC has been implicated in the pathogenesis of Crohn's disease, therefore the ability of *S. thermophilus* NCIMB 41856 to reduce the production of TNFα, the principle pro-inflammatory cytokine present in this condition, is highly important and warrants further investigation; inflammation-induced fibrosis leading to stricture formation in Crohn's disease represents a serious complication with important clinical implications [41]. reducing inflammation, firstly exerting its effects from the lumen of the gut and secondly, when intestinal barrier function is compromised, by crossing through the epithelium and interacting with the underlying cells. Here we have shown that this bacterium is capable of reducing epithelial cell death as well as the NF-κB and IL-8 response to pathogen, thereby.

In conclusion, *S. thermophilus* NCIMB 41856 is an iron-responsive probiotic strain of bacteria with far-reaching applications, capable of reducing egress of pathogenic bacteria from the lumen of the gut, improving barrier function and reducing inflammation. Clinical evaluation is now needed to determine whether its unique combination of effects *in vitro* translate to the treatment of IBD.

## Materials and Methods

### Ethics statement

Tissue was collected from patients after written informed written consent and with appropriate ethical approval from the Somerset Research Ethics Committee.

### Bacteria used

Species of lactic acid bacteria that have been employed as probiotics were used. These were: *L. bulgaricus* JB005, *L. casei* JB006, *L. casei* JB008, *B. animalis* JB007, and *B. bifidum* JB009 (isolated from yoghurt); *L. plantarum* JB012 and *L. helveticus* JB012 (isolated from probiotic capsules); and the commensal isolates *L. acidophilus* ASF360 and *L. salivarius* ASF361 (components of the Schaedler flora). Two strains of *S. thermophilus* (JB004 and NCIMB 41856) were isolated from yoghurt. Two strains of AIEC were used: HM427 and HM615 (kindly provided by Dr Barry Campbell and Prof Jon Rhodes, University of Liverpool), as was *E. coli* K12 and *E. coli* Nissle 1917 (isolated from Mutaflor (Ardeypharm GmbH, Herdecke, Germany)). All *E. coli* strains were grown in 10 ml volumes of LB broth (Oxoid, Cambridge, UK) at 37°C overnight. *S. thermophilus* was grown in M17 broth supplemented with lactose (Oxoid) and *Lactobacillus* spp were cultured in MRS broth (Oxoid) overnight in a microaerobic atmosphere. Lactic acid bacteria were cultured in serum-SAPI medium with and without 100 µM (-) noradrenaline (Sigma, Poole, UK) in order to determine if they were capable of responding to it. O.D. measurements were taken at 24, 48 and 72 hours in order to determine bacterial growth. Differences were analysed using a repeated-measures ANOVA.

### Proliferation and death of epithelial cells

All cell culture reagents were purchased from PAA Laboratories (Austria) unless otherwise specified. T84 and Caco-2 human adenocarcinoma cells (ECACC, Health Protection Agency Culture Collection, Salisbury, UK) were grown in DMEM/Ham's F-12 or DMEM respectively, supplemented with 10% FCS, 2 mM L-glutamine and 100 U/ml penicillin/streptomycin in 96-well tissue culture plates at an initial density of 2.4×10^4^ cells per well. After 3 days incubation, the medium was changed for one that was antibiotic-free and cells were labelled with BrdU in order to quantify proliferation. Bacteria were added to the epithelial cell cultures at an MOI of 30 and incubated for 24 hours at 37°C with 5% CO_2_. After 24 hours supernatants were harvested to determine cytotoxic effects of bacteria using the Cytotox 96 non-radioactive cytotoxicity assay kit (Promega, Southampton, UK) as directed by the manufacturer's instructions. Quantification of BrdU incorporation into the cells was determined using the cell proliferation biotrack ELISA system (GE Healthcare, Chalfont St Giles, UK) as per the manufacturer's instructions. Differences were analysed using paired *t*-tests (GraphPad Prism 5, California, USA).

### NF-κB assays

Caco-2 cells were seeded into 12-well tissue culture plates at an initial density of 6×10^5^ cells per well. After 2 days of culture, cells were transfected with a reporter plasmid having an NF-κB response element, pGL4.32 (Promega), and the internal control reporter pGL4.74 (Promega) using lipofectamine (Invitrogen, California, USA). 24 hours later, the medium was replaced with 1x HBSS (Invitrogen) and bacteria were added at an MOI of 30. After 40 hours, cells were lysed and luciferase activity was quantified using a Dual Luciferase Reporter Assay (Promega) as per the manufacturer's instructions. Differences were analysed using paired *t*-tests (GraphPad Prism 5). T84 cells could not be efficiently transfected using these reporter plasmids and therefore results are not shown.

### Detection of IL-8

In order to determine the effect of potential probiotic strains on IL-8 production T84 cells were seeded into 12-well tissue culture plates at an initial density of 6×10^5^ cells per well. After 3 days of culture, the medium was replaced with antibiotic-free medium and bacteria added at an MOI of 30. After 6 hours supernatants were harvested. Cytotoxicity was determined as above and production of IL-8 was quantified by ELISA using the human IL-8/CXCL8 DuoSet (R&D Systems, Minneapolis, USA) as per the manufacturer's instructions. IL-8 production was corrected for cell death and differences were analysed using paired *t*-tests (GraphPad Prism 5). Caco-2 cells did not produce sufficient levels of IL-8 and therefore results are not shown.

### Growth of epithelial cell monolayers and challenge with bacteria

Caco-2 and T84 cells were seeded onto 12-mm Transwell membranes (12 mm diameter, 3μm pore size; Corning Glass Works, Corning, NY) in 12-well tissue culture plates at an initial density of 3×10^5^ cells per insert. Plates were incubated at 37°C in 5% CO_2_ for 8–10 days until the cells formed confluent monolayers and the transepithelial resistance (TEER) was greater than 300 Ω/cm^2^ as measured with an epithelial voltmeter as an indicator of membrane permeability. The medium was then changed to one that was antibiotic free and bacteria were added to the apical well of the Transwell insert at an MOI of 30. TEER of all monolayers was measured at 2 hour intervals up to 12 hours and bacterial in the basal well were enumerated every 2 hours up to 10 hours. Differences were evaluated at each time point using paired *t*-tests (GraphPad Prism 5). In a separate experiment, medium was removed from the Transwells after 10 hours and the monolayers were examined as detailed below.

### Occludin staining

Transwell inserts were fixed in ice-cold methanol at 4°C overnight. Inserts were washed in PBS and the cells permeabilised with 0.1% Triton X-100 for 10 minutes before being washed again. Mouse anti-occludin monoclonal antibody (Zymed, California, USA) was diluted 1/200 and added to the apical chamber of the insert and incubated at room temperature for 45 minutes. Inserts were then washed in PBS and incubated for a further 45 minutes with TRITC-conjugated isotype-specific goat anti-mouse antibody (Southern Biotech, Birmingham, AL, USA) diluted 1/100 in the apical chamber. The inserts were then washed with PBS and the membranes were cut out of the inserts with a scalpel and mounted on slides with Vectashield containing DAPI (Vector Laboratories, California, USA). Fluorescent staining was examined on a Leica DMRA microscope equipped with a Hamamatsu Orca-ER monochrome camera. Ten fields of view per slide at 40x magnification were digitised using Leica Q-Fluoro software. Images were viewed using ImageJ software (http://rsb.info.nih.gov/ij) and positive pixels automatically counted as previously described [42]. The significance of differences was determined by one-way ANOVA (GraphPad Prism 5).

### T cell isolation and culture

Resected intestinal tissue was collected from patients undergoing surgery for complications associated with Crohn's disease or ulcerative colitis and from patients with colorectal cancer after informed written consent and with appropriate ethical approval (Somerset Research Ethics Committee). The mucosa was separated from the muscle, cut into small fragments and incubated in collagenase (100 U/ml; Sigma) for 2 hours at 37°C. Cells were washed in PBS and leukocytes purified over discontinuous Percoll gradients (35–75%; GE Healthcare). The cell count and viability was determined by trypan blue exclusion. Cells were resuspended to a final concentration of 5×10^6^/ml in RPMI 1640 supplemented with 10% FCS, 1 mM sodium pyruvate, 2 mM L-glutamine and 50μg/ml gentamicin and cultured on top of a type I collagen gel (PureCol; Nutacon BV, The Netherlands). It has previously been shown that co-culture of lamina propria T cells with ECM components prevents activation-induced apoptosis [43] and, in particular, ligation of β_1_ integrins [44]; type I collagen is used as a supporting material in order to allow leukocytes to survive and proliferate. Bacterial cell sonicates were added to each well at an equivalent concentration to an MOI of 30 and cells were cultured for 5 days at 37°C with 5% CO_2_. After 5 days leukocytes were liberated from the collagen gel by the addition of collagenase (1000 U/ml). Cells were washed and counted before RNA extraction.

### RT-qPCR

RNA was extracted from the cultured leukocytes using a Macherey-Nagel NucleoSpin RNA II Isolation Kit (ABgene, Epsom, UK). Synthesis of cDNA was carried out using 500 ng of random hexamers using the ImProm-II Reverse Transcription System (Promega) in a final volume of 20µl. All reactions were prepared according to the manufacturer's instructions giving a final magnesium chloride concentration of 3 mM. All cDNAs were diluted to a final volume of 100μl (1/5 dilution) using EB buffer (10 mM Tris HCl pH 8.4; Qiagen Ltd., Crawley, UK). Primers and probes were designed using Primer 3 (http://frodo.wi.mit.edu/primer3) and M-Fold using the human specific GenBank sequences for T-box21 (accession number NM_013351), GATA-3 (accession number NM_001002295), RORC (accession number NM_005060), Foxp3 (accession number NM_017009), IFNγ (accession number NM_000619), TNFα (accession number NM_000594), IL-4 (accession number NM_000589), IL-4δ2 (accession number NM_172348), IL-17A (accession number NM_002190), IL-10 (accession number NM_000572) and TGFβ (accession number NM_000660). The housekeeper gene hydroxymethylbilane synthase (HMBS) was used as an internal control. Quantitative PCR (qPCR) was performed using HotStarTaq Master Mix (Qiagen Ltd.). Gene specific amplification was performed using 0.2µM of each primer, 0.1µM of probe or SYBR Green 1 (1/100,000; Sigma) and 5μl of diluted cDNA in a final volume of 25μl. Magnesium chloride concentrations were adjusted to 4.5 mM in the final reaction by addition of 50 mM MgCl_2_. Sample incubations were performed in an MxPro3005P (Stratagene, California, USA) at 95°C for 15 minutes and then 45 cycles of 95°C for 15 seconds, and 60°C for 30 seconds during which the fluorescence data were collected. Data is expressed as the relative change in mRNA transcription following treatment and is normalised for cell number. No significant differences were seen between cells isolated from the three disease states and therefore results were pooled for analysis. Differences were analysed using paired *t*-tests (GraphPad Prism 5).

## Supporting Information

Figure S1 **Effect of probiotic on TEER in T84 (A) and Caco-2 cells (B).** * p≤0.05, ** p≤0.01 and *** p≤0.001.
(TIF)

Figure S2 **Percent change in cytokine mRNA levels in response to** ***E. coli*** **with and without probiotic.** Results are expressed as mean±S.E.M. * p≤0.05.
(TIF)
