# Supplementary figures and images for: Identification and Characterisation of an Iron-Responsive Candidate Probiotic
Source: PLoS One. 2011 Oct 19;6(10):e26507. doi: 10.1371/journal.pone.0026507 (PMC3198401; doi:10.1371/journal.pone.0026507)

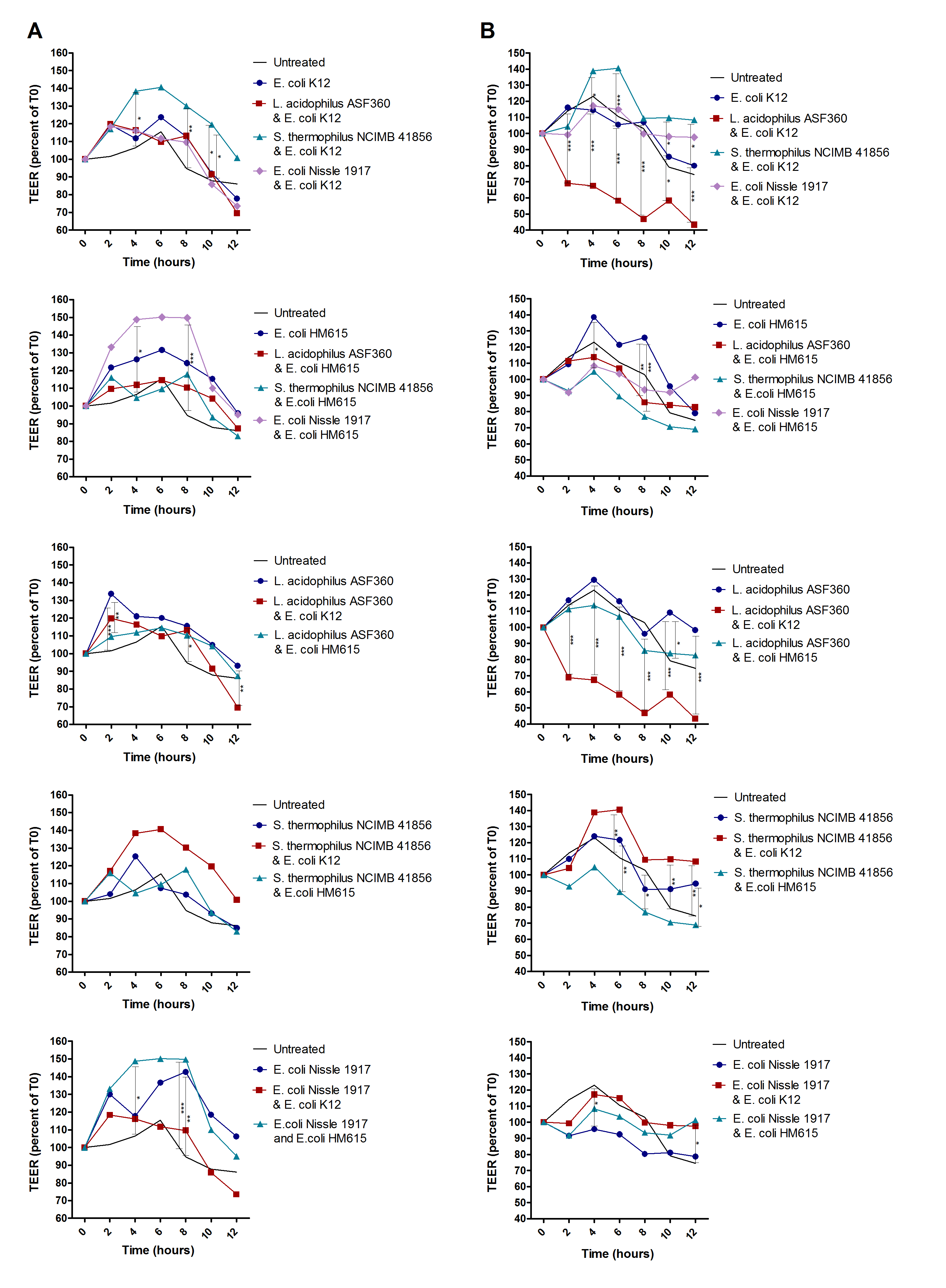

Supplement: Figure S1 — Effect of probiotic on TEER in T84 (A) and Caco-2 cells (B). * p≤0.05, ** p≤0.01 and *** p≤0.001. (TIF) [file pone.0026507.s001.tif]

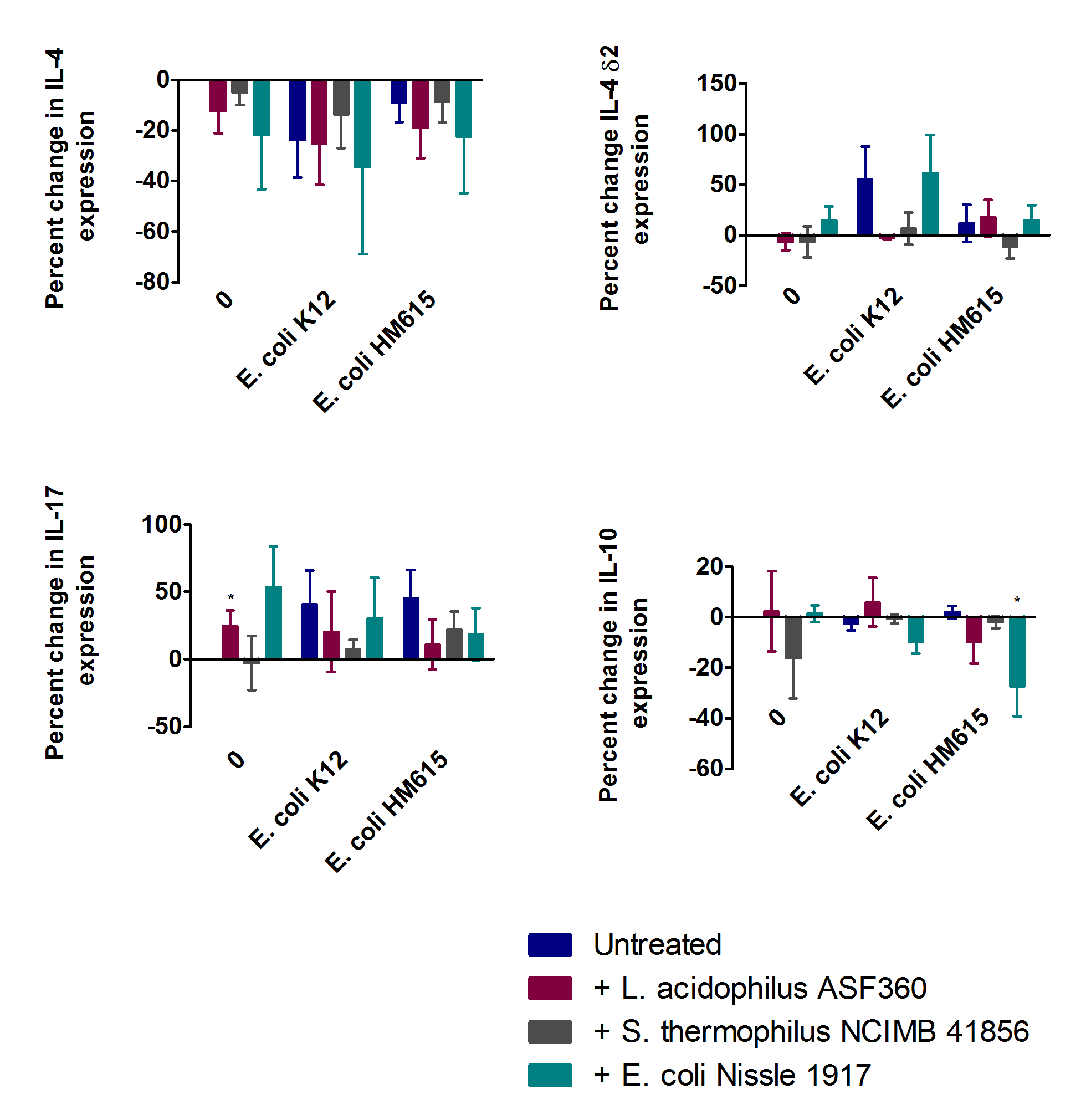

Supplement: Figure S2 — Percent change in cytokine mRNA levels in response to E. coli with and without probiotic. Results are expressed as mean±S.E.M. * p≤0.05. (TIF) [file pone.0026507.s002.tif]
